# Personal

**Published:** 1900-10

**Authors:** 


### PERSONAL.

HON. TIMOTHY L. WOODRUFF, president of the Maltine Company, of Brooklyn, has been renominated as Lieutenant-Governor of the State of New York. Governor Woodruff has already served two terms in that office and the compliment of a third nomination is unprecedented. Senator Depew in presenting Mr. Woodruff's name to the convention said: "He represents, in its best sense, the business-man in politics. He is the ideal young man in politics and meets all the requirements of the ever discussed question in the colleges of the educated man in politics. Beginning his career with no capital but brains, culture and courage, he started at the foot of the ladder, at small wages, and he achieved independence and won the confidence of the business community before he was thirty-five years old."

---

DR. JOHN H. GRANT, of Buffalo, has received from the State Civil Service Commission notice of the result of the examination held

September 1, 1900, for the position of Surgeon of the Soldiers' and Sailors' Home, at Bath, N. Y. His name stands No. 1 on the eligible list, which ought to entitle him to the appointment.

Dr. Grant is a veteran of the Civil War, and is at present a member of the Health Department staff of the city. He also served as a medical officer during the war with Spain.

---

Dr. Ernest Wende, Health Commissioner of Buffalo, was highly complimented recently in a letter received from A. W. Penniman, M.D., London, Eng., inquiring if it is possible for him to secure from 3,000 to 5,000 copies of a paper on infant mortality, read by Dr. Wende before the American Public Health Association. Dr. Penniman writes that the report contains a wealth of information upon the subject and that he has as yet seen nothing so concise and convincing.

---

Dr. I. N. Love, of St. Louis, editor of the *Medical Mirror*, announces his removal to New York, where he will make his home in the future. Dr. Love has been appointed a teacher in the department of internal medicine at the Post-Graduate Medical School and Hospital. New York is to be congratulated upon the accession to its professional ranks of so accomplished a physician, teacher and writer as Professor Love.

---

Maj. Charles K. Winne, Surgeon United States, Army has been assigned to duty at Fort Porter, Buffalo, N.Y., as surgeon of the post. Dr. Winne is a native of Buffalo and entered the army from here as assistant surgeon in 1861. He is the son of the late Dr. Charles Winne, who was a famous surgeon in his day, and a long time resident of Buffalo. Maj. Winne is welcomed here by a large circle of friends.

---

Dr. Matthew D. Mann, of Buffalo, read a paper before the Fort Dodge (Ia.) Medical Society, on the afternoon of August 14, 1900, entitled, Some common mistakes in gynecological practice. At the evening session the society also listened to a very instructive lecture by Professor Mann.

---

Dr. Wm. C. Krauss, of Buffalo, has been made one of the associate editors of the *Psychiatrische Wochenschrift*, published under the direction of Dr. J. Bresler, of Freiburg (Schlesien) Germany.

Dr. John Guiteras, who recently resigned the chair of pathology in the University of Pennsylvania to fill a similar position in the University of Havana, has established there a journal entitled, "Revista de Medicina Tropical."

Dr. S. Goldberg, of Buffalo, who has been traveling and studying in Europe for some time, as announced in the July issue of the Journal, has returned and resumed his professional work.

Dr. Harry Mead, of Buffalo, sailed September 18, 1900, for a tour in Europe. He will visit Paris, London and various countries of the continent and will be absent about two months.

Dr. Prescott Le Breton, of Buffalo, announces change of residence and office to 122 North Pearl Street. Office hours: 8-9 a.m., 1-2 p.m. and 6-7 p.m.

